# Social Skills Training for Adolescents With Autism Spectrum Disorder Using Facebook (Project Rex Connect): A Survey Study

**DOI:** 10.2196/mental.6605

**Published:** 2017-01-23

**Authors:** McLeod Frampton Gwynette, Danielle Morriss, Nancy Warren, James Truelove, Jennifer Warthen, Charles Paul Ross, George Mood, Charlotte Anne Snook, Jeffrey Borckardt

**Affiliations:** ^1^ Department of Psychiatry and Behavioral Sciences Medical University of South Carolina Charleston, SC United States; ^2^ Brown University Providence, RI United States; ^3^ Department of Psychology Loyola University of Maryland Baltimore, MD United States; ^4^ College of Charleston Charleston, SC United States; ^5^ Clemson University Clemson, SC United States; ^6^ Department of Anesthesia and Perioperative Medicine Medical University of South Carolina Charleston, SC United States; ^7^ Ralph H. Johnson VAMC Charleston, SC United States

**Keywords:** autism, social media, social skills

## Abstract

**Background:**

Adolescents with autism spectrum disorder (ASD) spend more time using electronic screen media than neurotypical peers; preliminary evidence suggests that computer-assisted or Web-based interventions may be beneficial for social skills acquisition. The current generation of adolescents accesses the Internet through computers or phones almost daily, and Facebook is the most frequently used social media platform among teenagers. This is the first research study to explore the use of Facebook as a therapeutic tool for adolescents with ASD.

**Objective:**

To study the feasibility and clinical impact of using a Web-based social platform in combination with social skills training for adolescents with ASD.

**Methods:**

This pilot study enrolled 6 participants (all males; mean age 14.1 years) in an online social skills training group using Facebook. Data was collected on the participants’ social and behavioral functioning at the start and conclusion of the intervention. Outcome measures included the Social Responsiveness Scale-2, the Social Skills Improvement System Rating Scale, and the Project Rex Parent Survey. Participants were surveyed at the conclusion of the intervention regarding their experience.

**Results:**

No statistically significant differences in measurable outcomes were observed. However, the online addition of Facebook was well received by participants and their parents. The Facebook intervention was able to be executed with a careful privacy protocol in place and at minimal safety risk to participants.

**Conclusions:**

The utilization of Facebook to facilitate delivery of social skills training for adolescents with ASD appears to be feasible, although the clinical impact of such an addition is still unclear. It is important to note that social difficulties of participants persisted with the addition of the online platform and participants still required assistance to engage with peers in an online environment. A Web-based intervention such as the one utilized in this study has the potential to reach a mass number of patients with ASD and could address disparities in access to in-person treatment services. However, the complexity and evolving nature of Facebook’s website and privacy settings leads to a number of unique online safety concerns that may limit its clinical utility. Issues encountered in our study support the development of an alternative and closed Web-based social platform designed specifically for the target audience with ASD; this platform could be a safer and more easily moderated setting for aiding in social skills development. Despite a small sample size with no statistically significant improvements of target symptoms, the use of electronic screen media as a therapeutic tool for adolescents with ASD is still a promising area of research warranting further investigation. Our study helps inform future obstacles regarding feasibility and safety.

## Introduction

Autism spectrum disorder (ASD) affects 1 in 68 children in the United States and is characterized by deficits in social communication; impaired social interactions; and repetitive and/or restricted interests, behaviors, and activities [1,2]. Adolescence is a time of particular risk and opportunity for patients and families affected by ASD, as the presence or absence of effective interventions can profoundly influence a successful transition into adulthood. These services often include group social skills training (SST), an intervention with demonstrated effectiveness in improving core ASD symptoms and social deficits in high-functioning adolescents [3-6].

Currently, SST groups are run in person, usually weekly over a 2-4-month period. However, access to traditional SST programs is limited, mirroring existing gaps for other ASD services that are often related to geographic and socioeconomic disparities [7]. Multiple published studies of interventions using Facebook have demonstrated the social media platform’s capability to reach other underserved patient populations [8-10], indicating that Facebook may also be a viable modality for reaching patients with ASD.

Interventions utilizing Facebook technology have been demonstrated to be effective in treating numerous other mental health conditions [11-19]. A recent study found 92% of US teens go online daily [20] and 71% of teens ages 13-17 use Facebook [21]. Research of adolescents with ASD reflects a similarly high amount of use [22], so Web-based care has the potential to be desirable to adolescents with ASD, which could improve interest or time spent utilizing a therapeutic tool. Studies of social media use by adolescents with ASD also demonstrate they most often interact online with acquaintances they previously met in person [23] and may improve social interactions [22], leading to support of its use in conjunction with SST.

For these reasons, the investigators hypothesized that utilization of Facebook to deliver SST for adolescents with ASD would be feasible and acceptable to participants and possibly improve clinical outcomes. We define feasibility as a capability of being executed and we define acceptability as favorable ratings on participant satisfaction surveys. This pilot study is unique as there are no other published studies to date that augment face-to-face social skills instruction with a social networking site (SNS) component or curriculum.

## Methods

### Participants

Participants were recruited offline from within the Project Rex clinic, an SST program at the Medical University of South Carolina serving adolescents with ASD along with their families [24]. A survey of Project Rex parents indicated most would be interested in having their child use a Web-based social network to build social skills and keep in contact with friends. Enrolled participants consisted of 6 males ranging from 12 to 19 years of age (mean 14.1 years), with a previous diagnosis of ASD and an IQ in the normal range.

Pre- and posttreatment surveys of participants, parents, and teachers (or other responsible adult) were administered via REDCap [25], a Web-based survey and data collection platform. Outcome measures included the Social Responsiveness Scale-2 (SRS-2) [26]; the Social Skills Improvement System Rating Scale (SSiS-RS) [27]; the Project Rex Parent Survey, a 12-item Likert scale measuring utilization of various social skills in the past 2 weeks; and the Project Rex Connect Participant Survey, a 10-item *yes/no* survey on the participants’ opinions about the treatment received.

### Procedures

Participants were recruited from the Project Rex clinical program and were followed for a period of 8 weeks. The participants were enrolled in a closed, invite-only *group* within Facebook facilitated by the investigators for 8 weeks. Online groups within Facebook provide a platform where users can post comments, pictures, and videos and have discussions. Each participant was directly aided by investigators to set up their account with a deidentified username and specific privacy settings. Participants were previously acquainted with each other from prior participation in the live Project Rex SST, which they had completed 2 weeks prior to study enrollment. The Facebook group curriculum included weekly shared topics and encouraged the use of social skills taught in the Project Rex SST, such as starting conversations, exchanging information with peers, and giving compliments.

**Figure 1:**
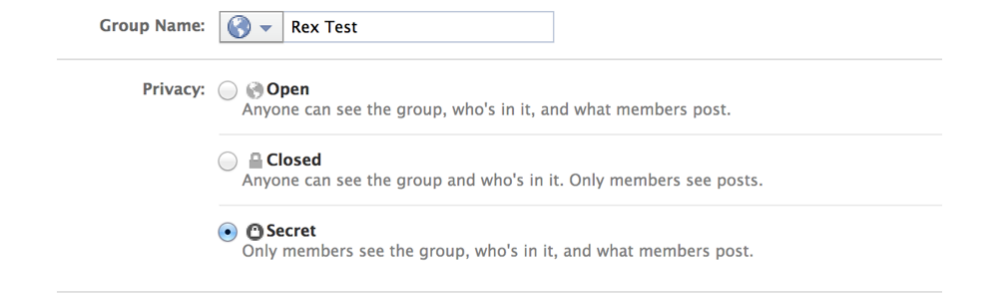
Screenshot of Facebook group privacy setting utilized in this study.

**Figure 2:**
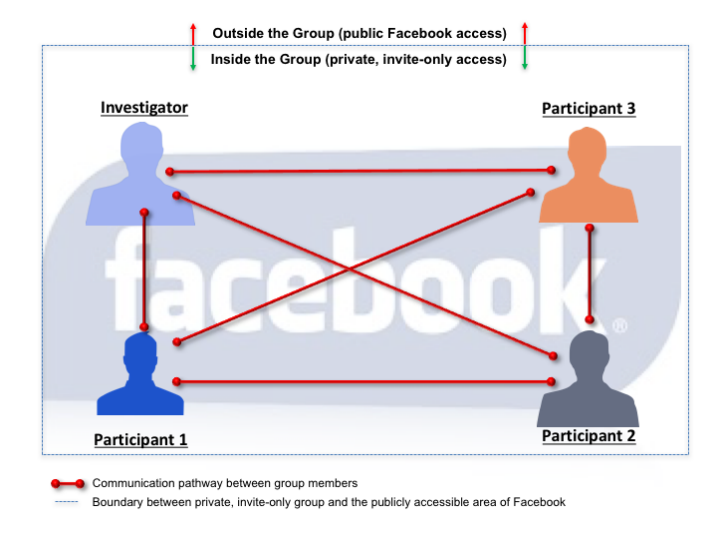
Overview of the secret, private Facebook group utilized in this study.

To maximize the likelihood of safe and appropriate Facebook use, participants and their parents were provided with education and training on use of the account, online etiquette, and maintenance of privacy settings. Inappropriate use included adverse messaging, inappropriate friend requests, and cyberbullying. The participants were informed of the study rule that any sharing of photos or videos from a private collection or from the Internet would require parental approval prior to the participant posting it. All group settings were placed on Facebook’s option of *secret group* to minimize the risk of a breach in confidentiality (see Figure 1), which meant information posted by participants was not publicly available. Participants were also unable to invite new group members due to privacy settings. Investigators and participants were Facebook *friends* with one another and no one else, further limiting access of individuals outside the study (see Figure 2).

The participants were free to use Facebook as they desired within the study guidelines. In an attempt to encourage social engagement, participants were asked to log onto their accounts for a minimum of 6 days a week with a minimum time frame of 15 minutes per Facebook session. The investigators provided a weekly topic of discussion and also posted general reminders drawn from the Project Rex curriculum on how to initiate and maintain a conversation.

Following safety protocols, a study investigator reviewed all posts on a daily basis. Investigators maintained sole access to each account’s email address and had shared access to passwords, allowing access to intervene if any participant posted inappropriate material or attempted to obtain friends outside of Project Rex. Due to account settings, Facebook would automatically send an email if there were any attempts at changing the account password or accepting friend requests from outside members. All participant email accounts were checked on a daily basis.

## Results

Upon the conclusion of the study, there were no statistically significant differences in pretreatment and posttreatment measures according to the parent- and teacher-completed SRS-2; the parent-, teacher-, and participant-completed SSiS-RS; or the Project Rex Parent Survey. Outcome measures of this study were limited by the small sample size. Results of the participant experience survey indicate high satisfaction and acceptability rates. A total of 80%-100% of participants (N=5) selected “yes,” indicating complete satisfaction, for each item. One participant did not complete the participant survey (see Table 1).

**Table 1:** Results of the participant satisfaction survey (N=5).

Question	Participants replying “yes,” n (%)
I enjoyed my participation in this group.	4 (80)
I practiced introducing myself to others in this group.	4 (80)
I practiced my “starting a conversation” skills in this group.	5 (100)
I practiced my listening skills in this group.	4 (80)
I practiced my “asking a question” skills in this group.	4 (80)
I practiced my “having a conversation” skills in this group.	4 (80)
I believe my social skills improved from my participation in this group.	5 (100)
I believe I have made friends from this group experience.	4 (80)
I will keep in contact with the other group members.	4 (80)
I would repeat this group.	4 (80)
I would recommend the Project Rex program to my peers or friends.	4 (80)

There were no adverse events during the course of this study, including breaches of confidentiality, cyberbullying, or inappropriate contact with individuals outside of the Facebook group. One participant did join a fan club and required multiple redirections, twice by Facebook message and once by telephone, in order to comply with the study protocol. Participants tolerated the intervention well and they all completed the study protocol.

## Discussion

This is the first research study to explore the use of Facebook as a therapeutic tool for adolescents with ASD. The results of this pilot study show that the utilization of Facebook to facilitate delivery of social skills training for adolescents with ASD appears to be feasible, although the clinical impact of such an addition is still unclear. Our study was conducted safely and confidentially, with the execution of many privacy measures not typical of community Facebook use. While there were no statistically significant effects of the Facebook intervention, it is very likely that this pilot study was significantly underpowered by having only 6 participants.

There were several interesting observations gathered from this pilot study that should influence follow-up studies of a similar nature. Investigators found participants’ impaired social functioning was observable even in an online environment. That is, computers and Web-based social media did not *mask* the symptoms of ASD in this study. Future studies could potentially begin with a basic training session aimed at the participants’ core deficits and ways to generalize skills acquired from the live groups to the online environment. For example, investigators noted participants in the Facebook group frequently posted material of interest to them, but not necessarily to others. In future studies, it may be beneficial to teach participants to consider what others may be interested in viewing. In this way, researchers could potentially address deficits in the ability of individuals with ASD to experience the world through the perspective of another person, a core deficit related to “theory of mind” [28,29]. Although not utilized in this study, collecting data on the frequency and quality of participants’ posts while online could help further characterize patterns of social media usage in individuals with ASD, assist in measuring potential response to Web-based social skills training, and serve as a useful feedback tool for the participants. Also not investigated in this study was observer-expectancy effect. It is likely that investigators’ monitoring techniques had some effect on subjects’ behavioral reactivity, although whether this positively or negatively impacted subject satisfaction and participation warrants further exploration.

The privacy restraints used in this study represent a potential trade-off between the engagement and use of Facebook by the participants and security and confidentiality. The privacy restrictions allowed participants to have a more controlled and supported online experience versus the opportunity to practice newly acquired skills in a more anonymous virtual platform. Given the extensive privacy features used during this study, Facebook’s evolving privacy policies, and the utility of having a closed system designed as an adjunct for SST, it may be beneficial to develop a new, self-contained SNS geared specifically toward ASD youth and adults. This would provide the opportunity for more secure interactions between participants as it would lessen the risk of *public* posting of information that could happen with Facebook use. If a private site was utilized, investigators would also have increased ability to moderate the online environment if inappropriate posting did occur.

A direction suggested by multiple participants was a gaming component of the online experience. It may be beneficial from an engagement standpoint to incorporate gaming into an online social group for patients with ASD. Evidence suggests patients with ASD most frequently visit websites related to gaming [22] and the potential research directions for therapeutic gaming is largely unexplored. This would represent another benefit of developing a self-contained SNS geared specifically toward ASD youth and adults, as a gaming component could be specifically targeted to maximize opportunities for social interaction.

Given its potential to reach a vast number of patients with ASD, especially those who are underserved, the feasibility and effectiveness of using an SNS to enhance social functioning warrants further investigation. Potential benefits of adding such a component include the increased ability to engage and attract youth with ASD and that the nature of social networking allows for an extended reach to additional populations who may not typically be able to access SST. Ideally, an SNS platform could be used to reinforce new friendships established with SST peers and serve as a virtual training ground or “dress rehearsal” for the development of live friendships outside of the clinical realm. If successfully implemented, an SNS platform can be a powerful modality both for the delivery of SST and for addressing disparities in access to clinical services for patients and families affected by ASD.

## Abbreviations

ASD: autism spectrum disorder
SNS: social networking site
SRS-2: Social Responsiveness Scale-2
SSiS-RS: Social Skills Improvement System Rating Scale
SST: social skills training
